# Development and validation of a preoperative model for predicting positive proximal margins in adenocarcinoma of the esophagogastric junction and assessing safe margin distance

**DOI:** 10.3389/fonc.2024.1503728

**Published:** 2024-12-10

**Authors:** Zhenjiang Guo, Ning Wang, Guangyuan Zhao, Liqiang Du, Zhaobo Cui, Fangzhen Liu

**Affiliations:** ^1^ Department of Gastrointestinal, Hengshui People’s Hospital, Hengshui, Hebei, China; ^2^ Department of Respiratory and Critical Care Medicine, Hengshui People’s Hospital, Hengshui, Hebei, China

**Keywords:** esophagogastric Junction, adenocarcinoma, advanced, positive proximal margin, predictive model

## Abstract

**Objective:**

To develop and validate a model for preoperative prediction of positive proximal margins for adenocarcinoma of the esophagogastric junction (AEG) by transabdominal approach, and to analyze the safe margin distances for patients with different risks of positive proximal margins.

**Materials and methods:**

A retrospective analysis was performed on 284 AEG patients who underwent surgery via the transabdominal approach at Hengshui People’s Hospital between January 2017 and December 2023. Patients were divided into a training set (*n*=201, first five years) and a test set (*n*=83, last two years). Clinicopathologic factors potentially influencing margin status were collected. The synthetic minority oversampling technique (SMOTE) was applied to address class imbalance in the training set. Two nomogram models were developed: one based on the original training set and the other using the SMOTE dataset. The model’s performance was compared using the test set, with the area under the curve (AUC) used to evaluate discrimination and the Hosmer-Lemeshow test used for model fit. The best-performing model was used to calculate total scores for the entire cohort, and the optimal cutoff value was determined via the ROC curve. Patients were classified into low- and high-risk groups based on the total score, and optimal margin distances were determined using Youden’s index.

**Results:**

The model developed using the SMOTE dataset showed superior AUC for predicting positive proximal margins in the test set compared to the model based on the original training set (0.814 *vs.* 0.780). Independent predictors of positive proximal margins included Borrmann classification, Lauren classification, cT stage, tumor differentiation, and Siewert classification. The Hosmer-Lemeshow test showed a good model fit (χ² = 5.397, *P* = 0.612). Using a cutoff total score of 206.811, patients were divided into low-risk (score < 206.811) and high-risk (score ≥ 206.811) groups, with an AUC of 0.788. For the low-risk group, a proximal margin distance of 2.75 cm yielded an AUC of 0.824, with a sensitivity of 54.5%, specificity of 97.9%, and a Youden’s index of 0.524. For the high-risk group, a margin distance of 3.85 cm provided an AUC of 0.813, sensitivity of 73.1%, specificity of 80.0%, and a Youden’s index of 0.531.

**Conclusions:**

The nomogram may offer a valuable preoperative tool for assessing the risk of positive proximal margins in AEG patients. While it holds the potential to inform surgical decision-making and help determine appropriate margin distances, further validation in larger and more diverse cohorts is needed to confirm its clinical utility.

## Introduction

The incidence of adenocarcinoma of esophagogastric junction (AEG) is on the rise worldwide, and its unique anatomical location and biological characteristics have made it a focal point of clinical research (1–3). Surgical resection remains the primary treatment for AEG patients, yet there is no consensus regarding the optimal surgical approach, lymph node dissection, or perioperative treatment (4–6). The Siewert classification is the predominant system used to guide surgical planning for AEG: typically, Siewert type I is treated via a transthoracic approach, while Siewert type III is approached transabdominal through the esophageal hiatus. Siewert type II remains controversial, with both transthoracic and transabdominal approaches considered (7, 8).

A notable randomized controlled trial (JCOG 9502) from Japan compared the outcomes of transhiatal surgery with a left thoracoabdominal approach in Siewert type II/III patients with ≤3cm esophageal invasion. The study found no significant difference in long-term survival between the groups; however, the left thoracoabdominal approach led to more postoperative complications. Consequently, a transhiatal approach is recommended for patients with ≤3cm esophageal involvement (9).

While the transhiatal approach has certain benefits, it is associated with a shorter free esophageal length, which increases the risk of positive proximal margins. Positive margins, in turn, can lead to incomplete tumor resection and a worse prognosis (10). Studies have identified several risk factors for positive margins, including tumor size, depth of invasion, Borrmann classification, degree of differentiation, Lauren classification, and margin distance. A preoperative evaluation of these risk factors is essential to guide surgical decision-making (11, 12). Rompen IF et al. (13) examined the impact of proximal margin distance (PMD) in distal gastrectomy for gastric adenocarcinoma. Among 176 patients, 39.8% had adequate PMD. While PMD didn’t affect survival in intestinal patients, it improved outcomes for diffuse-type patients. The study highlights the importance of adequate surgical margins, suggesting less extensive resections for intestinal types. China expert consensus guidelines recommend maintaining a margin distance of ≥3cm in Siewert type II/III AEG patients with cT2 or greater staging. Rapidly frozen pathology is suggested for patients at high risk of positive margins (14, 15). However, relying solely on cT staging to determine margin distance is inadequate, especially for those with submucosal invasion or skip metastasis (16). Moreover, with the application of total laparoscopic anastomosis techniques, patients with positive margins indicated by intraoperative frozen pathology may technically be unable to undergo multiple extensive resections. Altering the surgical approach during the procedure further increases patient trauma and surgical risks.

To the best of our knowledge, there is currently no predictive model for diagnosing positive surgical margins in gastric cancer or AEG. This study aims to develop and validate a preoperative predictive model for proximal margin positivity in AEG patients, analyzing safe margin distances for patients at varying risks of positive margins to reduce the incidence of positive margins.

## Materials and methods

This prediction model study is reported by the TRIPOD checklist (17).

### Study population

Clinicopathologic data were collected from AEG patients who underwent radical resection at Hengshui People’s Hospital between January 2017 and December 2023. The inclusion criteria were as follows (1): pathologically confirmed AEG (2); esophageal invasion length < 3cm (3); no preoperative treatment (4); transabdominal approach with no residual tumor (5); clear histopathological determination of invasion status at the proximal margin. The exclusion criteria included (1): emergency surgery (2); multifocal gastric cancer (3); the presence of other active tumors. All surgeries were performed by a dedicated surgical team. The study received approval from the Ethics Committee of Hengshui People’s Hospital (Ethics No. 2022-2-015), and informed consent was obtained from the patients or their families.

### Candidate variables

Data on age, gender, tumor size, Siewert staging, Borrmann staging, tumor differentiation, Lauren staging, cT staging, and cN staging were collected from the patients. Siewert type II refers to tumors with their center located between 1 cm and 2 cm below the esophagogastric junction. In comparison, Siewert type III pertains to tumors positioned between 2cm and 5cm below the esophagogastric junction. The margin distance is defined as the distance from the esophageal incision margin to the upper margin of the tumor, measured intraoperatively. Since the margin distance can only be determined during surgery, it was not included as a predictive variable in the model. After the predictive model was developed, the optimal cut-off value for a safe margin distance was determined through ROC curve analysis.

### Diagnostic criteria for positive proximal margins

Positive proximal margins were defined as the presence of residual tumor cells located within 1 mm from the esophageal margins under microscopic examination. In contrast, margins were classified as negative if no tumor cells were found within this distance.

### Development and validation cohorts: approaches for addressing imbalanced data

Patients who met the criteria during the study period were included in the study cohort (*n*=284). The patients from the first 5 years and the last 2 years of the study were designated as the training set (*n*=201) and test set (*n*=83), respectively. The training set was utilized to fit the model, while the test set was employed to evaluate the model’s predictive performance. In this study, the positive samples in the training set consisted of patients with positive margins (*n*=26). In contrast, the negative samples included patients with negative margins (*n*=175), resulting in a significant imbalance between positive and negative samples. To address this imbalance, a new SMOTE dataset (*n*=208) was generated based on the samples in the training set using the Synthetic Minority Oversampling Technique (SMOTE), resulting in 104 positive and 104 negative samples (18). No statistical techniques to handle missing data were needed in either cohort.

### Comparison of the prediction performance of the two prediction models

Nomogram prediction models were constructed using the original training set and the SMOTE dataset. The prediction performance of these two models was then compared using the test set. Both models utilized the Akaike Information Criterion (AIC) stepwise regression method to select independent predictor variables and build logistic regression models. A lower AIC value indicates a better model fit (19). The area under the curve (AUC) of the ROC curve was employed to evaluate the discriminative ability of the two models in the test set, while the fit curve and the Hosmer-Lemeshow test were used to assess the overall fit of the constructed models.

### Risk stratification of positive proximal margin and safe margin distance

The model demonstrating superior predictive performance was selected to calculate the total score for the study cohort. The ROC curve was used to determine the optimal cutoff value of the total score for predicting positive proximal margins in AEG. Based on this, the cohort was divided into low-risk and high-risk groups for proximal margin positivity. The ROC curve was then applied again to calculate the safe margin distance between these two groups. The threshold for the margin distances was subsequently analyzed, with the value that maximized Youden’s Index chosen as the optimal cutoff.

### Statistics analysis

Data analysis was conducted using SPSS 22.0 and R 4.2.3 software. Categorical variables were expressed as frequency rates, with comparisons made using the chi-square test. The DMwR package in R was utilized for SMOTE data processing, while the stepAIC function from the MASS package was employed to perform multivariate logistic regression. The rms package was used to construct the nomogram model, and the pROC package facilitated the plotting of the ROC curve and the calculation of the area under the curve (AUC). The goodness of fit was assessed using the Hosmer-Lemeshow test and calibration curves. Additionally, the nomogramFormula package was used to calculate the total score for the model. A *p*-value of ≤ 0.05 was considered statistically significant.

## Results

### Basic characteristics of the study population

A total of 284 patients were included in this study, comprising 175 males and 109 females. The median age was 63 years, with an age range of 34 to 76 years. 37 patients were classified in the proximal margin positive group, while 247 patients were in the margin negative group. A comparison of the clinicopathologic characteristics between these two groups is detailed in **Table 1**. The incidence of positive proximal margins was found to be 13.03% (37/284).

**Table 1:** Preoperative baseline characteristics of 2 groups of patients with AEG with different proximal margin status [*n* (%)].

Variant	Proximal margin positive group (*n*=37)	Proximal margin negative group (*n*=247)	χ2 value	P-value
Age (years)			0.371	0.543
<65	18 (48.6%)	107 (43.3%)		
≥65	19 (51.4%)	140 (56.7%)		
Gender			2.603	0.107
male	23 (62.2%)	152 (61.5%)		
female	14 (37.8%)	95 (38.5%)		
Tumor size(cm)			0.170	0.680
<4	21 (56.8%)	149 (60.3%)		
≥4	16 (43.2%)	98 (39.7%)		
Siewert type			6.041	0.014
II	8 (21.6%)	21 (8.5%)		
III	29 (78.4%)	226 (91.5%)		
Borrmann type			6.935	0.008
I/II	10 (27%)	124 (50.2%)		
III/IV	27 (73%)	123 (49.8%)		
Tumor differentiation			4.840	0.028
well-moderately	12 (32.4%)	128 (51.8%)		
poorly-undifferentiated	25 (67.6%)	119 (48.2%)		
Lauren type			15.324	<0.001
intestinal	12 (32.4%)	163 (66.0%)		
diffuse/mixed	25 (67.6%)	84 (34.0%)		
cT staging			6.351	0.012
T2-3	15 (40.5%)	154 (62.3%)		
T4	22 (59.5%)	93 (37.7%)		
cN Staging			5.459	0.019
N0	14 (37.8%)	144 (58.3%)		
N+	23 (62.2%)	103 (41.7%)		

### Comparison of the predictive performance of two prediction models

**Figure 1:**
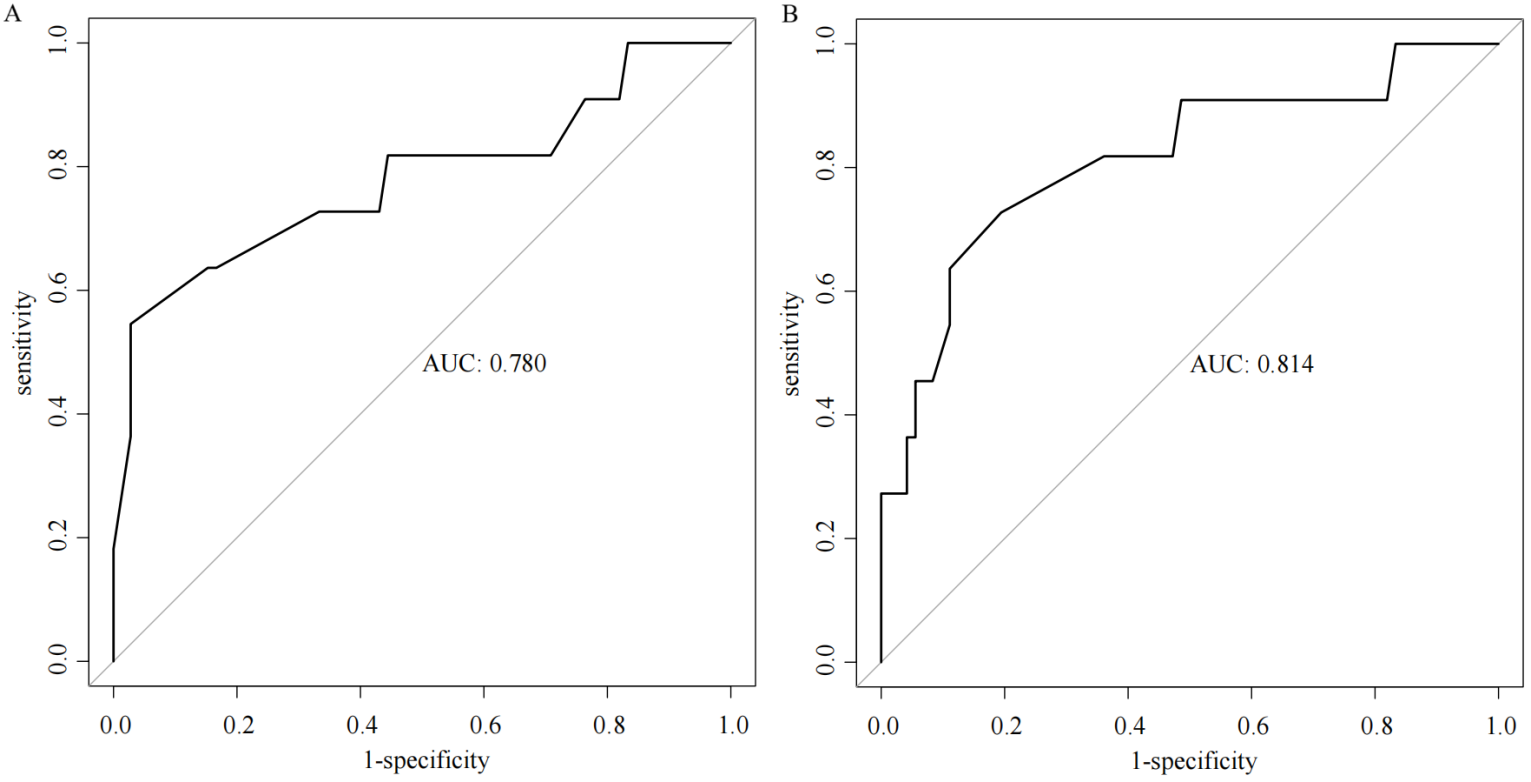
ROC curves for two prediction models [**(A)** original training set; **(B)** SMOTE dataset].

Multiple stepwise regression analysis of the predictor variables from the training set was conducted using the stepAIC function. The results indicated that the model achieved the smallest AIC value (134.03) when incorporating Borrmann staging, Lauren staging, cT staging, and tumor differentiation. In contrast, analysis of the SMOTE dataset revealed that the model yielded a smaller AIC value (229.02) when including Borrmann staging, Lauren staging, cT staging, tumor differentiation, and Siewert staging. The AUC value of the model based on the SMOTE dataset in predicting positive proximal margins in the test set was higher than that of the original training set (0.814 *vs.* 0.780), indicating superior predictive performance, as illustrated in **Figure 1**.

### Validation of the prediction performance of the smote dataset model

**Figure 2:**
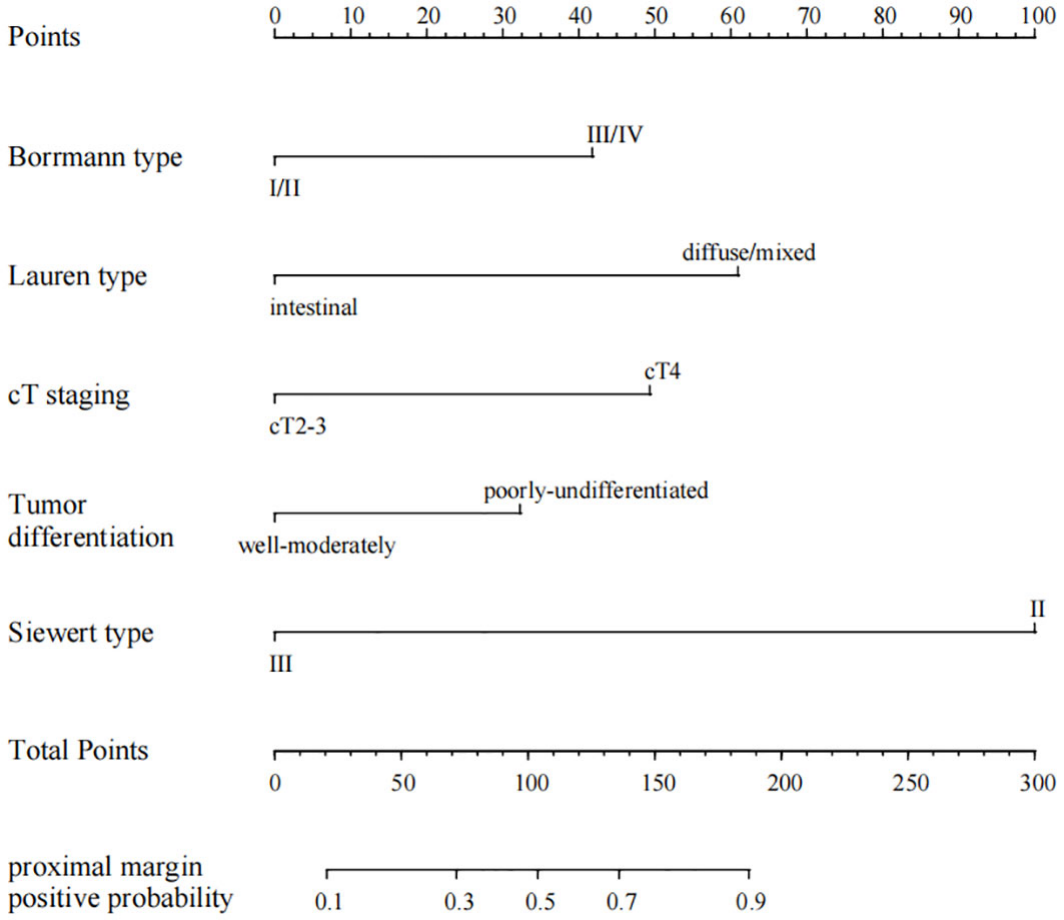
Nomogram of the positive proximal margin prediction model for AEG patients based on the SMOTE dataset.

**Figure 3:**
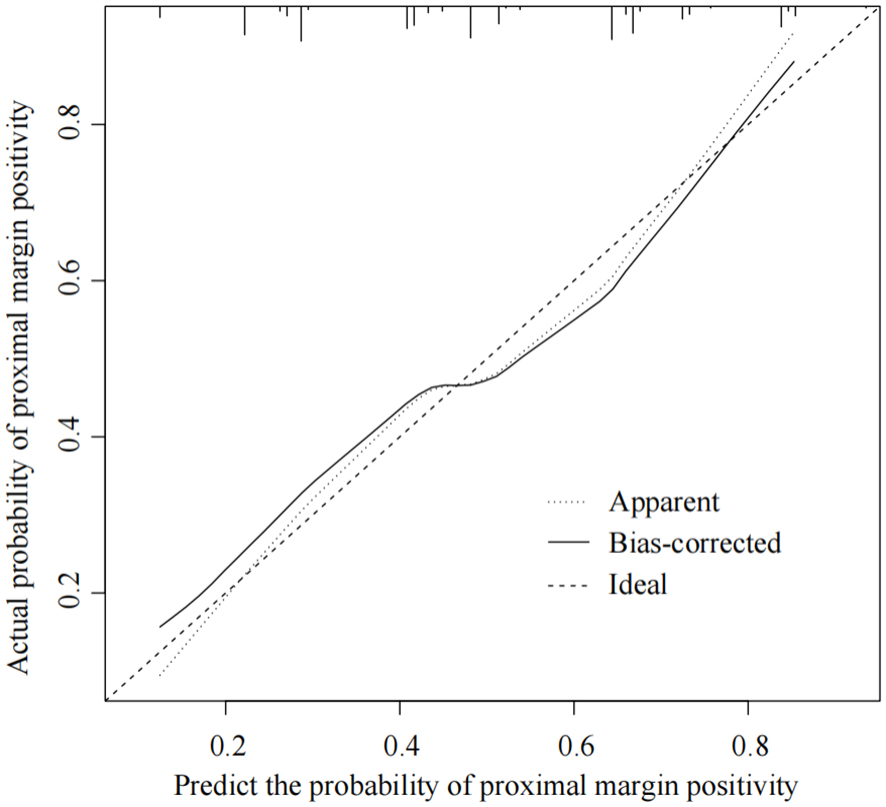
Calibration curves of the nomogram prediction model in the test set.

Multivariate logistic regression analysis identified Borrmann staging, Lauren staging, cT staging, tumor differentiation, and Siewert staging as independent predictors of proximal margin positivity within the SMOTE dataset (all *P* < 0.05), as shown in **Table 2**. A nomogram prediction model for AEG proximal margin positivity, which included the five predictors, was established (**Figure 2**). The online calculator available at https://guozhenjiang01.shinyapps.io/DynNomapp/ can be used to facilitate practical modeling. The calibration curve demonstrated a good fit between the predicted probabilities of AEG proximal margin positivity and the actual occurrence in the test set (**Figure 3**). Additionally, the Hosmer-Lemeshow test indicated a satisfactory goodness-of-fit (*χ* ^2^ = 5.397, *P* = 0.612).

**Table 2:** Multivariate Logistic regression analysis of positive proximal margin of AEG.

Variant	*β*	*SE*	*Wald χ^2^*	*OR* (95% *CI*)	*P*
Age (≥65 years *vs.* <65 years)	-0.314	0.332	0.893	0.731 (0.381-1.401)	0.345
Gender (female *vs.* male)	-0.451	0.501	0.813	0.637 (0.239-1.699)	0.367
Tumor size (≥4cm *vs*. <4cm)	-0.395	0.374	1.118	0.674 (0.324-1.401)	0.290
Siewert type (type II *vs.* type I)	1.172	0.469	6.239	3.227 (1.287-8.094)	0.012
Borrmann type (type III/IV *vs*. type I/II)	0.701	0.343	4.176	2.015 (1.029-3.947)	0.041
Tumor differentiation (well-moderately *vs*. poorly-undifferentiated)	1.317	0.349	14.209	3.731 (1.882-7.400)	0.000
Lauren type (diffuse/mixed *vs*. intestinal)	1.155	0.358	10.408	3.173 (1.573-6.399)	0.001
cT staging (cT4 *vs*. cT2-3)	0.678	0.333	4.153	1.969 (1.026-3.779)	0.042
cN staging [cN(+) stage *vs*. cN0 stage]	0.478	0.331	2.085	1.613 (0.843-3.085)	0.149
constant	-1.830	0.693	6.982	0.160	0.008

### Evaluation of safe surgical margins for patients with high and low risk of positive margins

**Figure 4:**
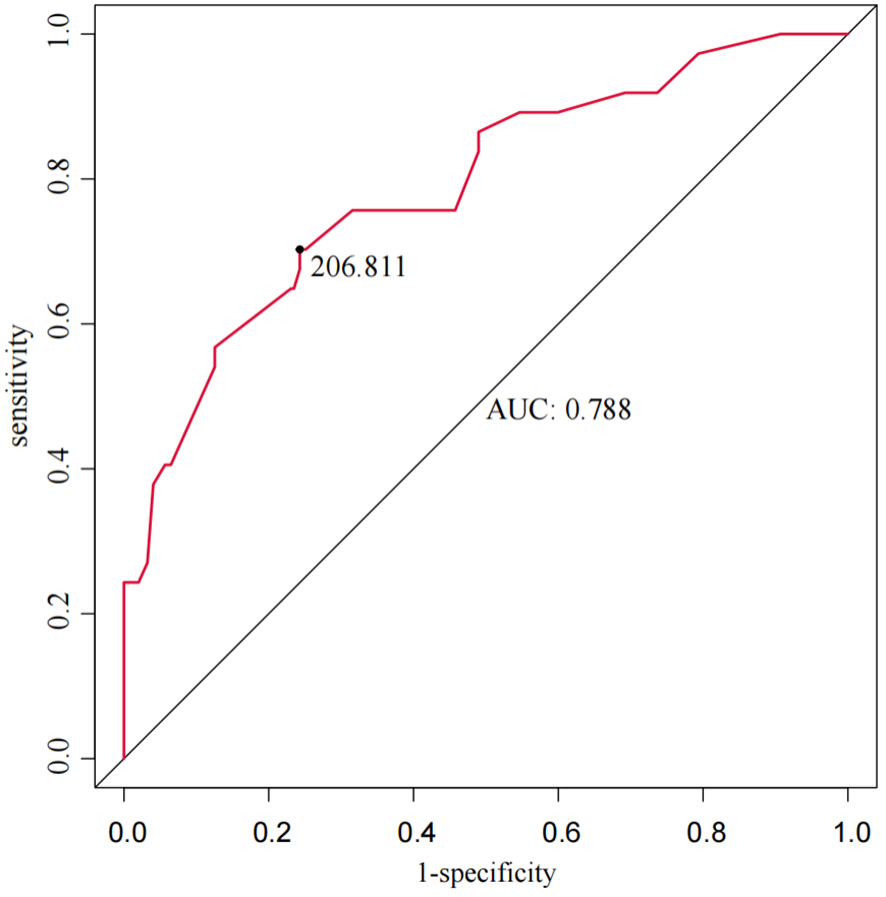
Total score predicts ROC curves for positive proximal margins in AEG patients.

**Figure 5:**
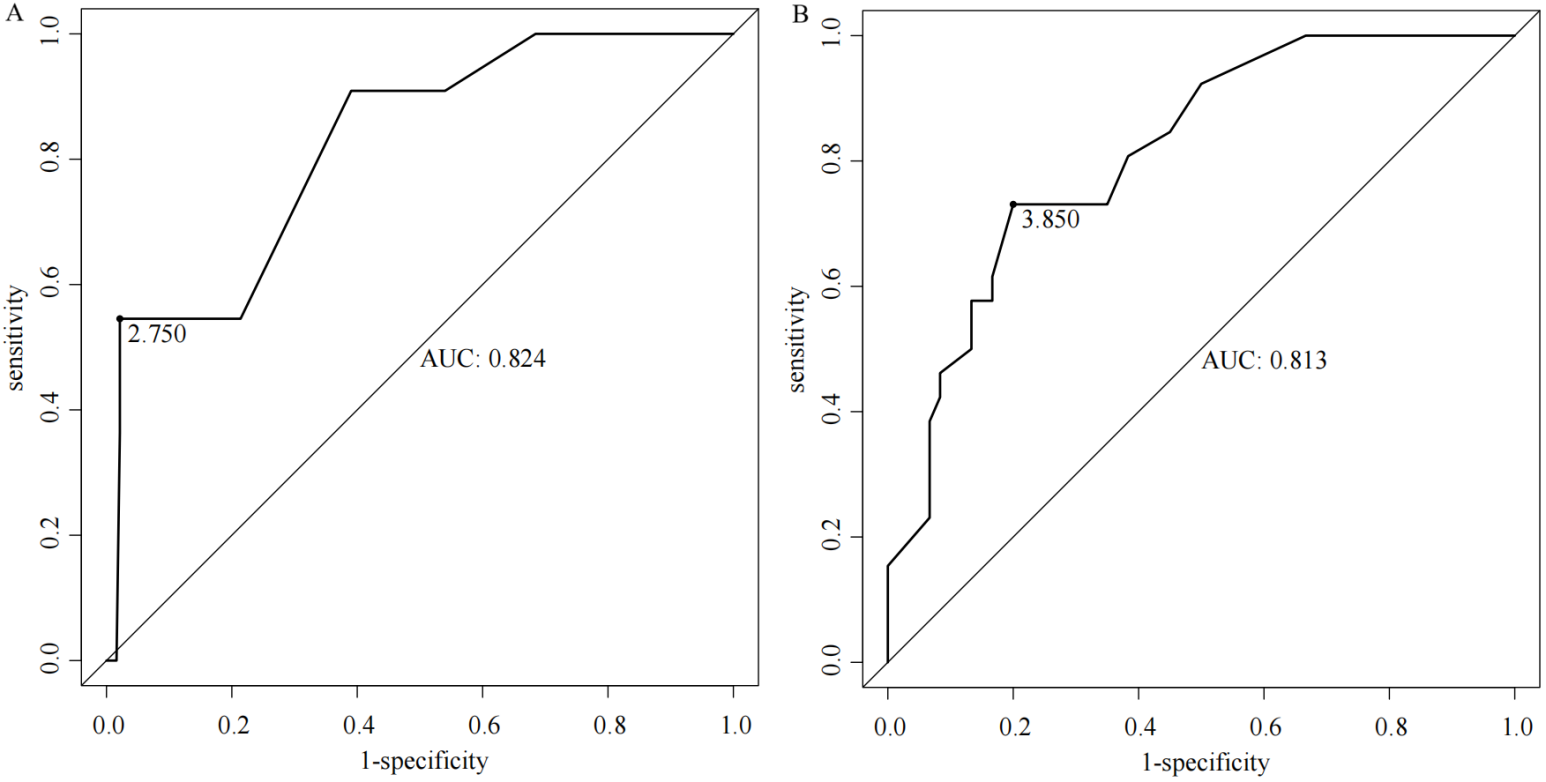
ROC curves of margin distance predicting the risk of positive proximal margins in patients with different AEGs [**(A)** low-risk group; **(B)** high-risk group].

The total score for the entire study cohort was calculated based on the nomogram model derived from the SMOTE dataset. This involved comparing the scores of each of the five predictor variables and summing these to obtain a total score. The optimal cutoff value for predicting AEG proximal margin positivity was identified through the ROC curve analysis, yielding a total score of 206.811. This score allowed for the categorization of patients into a low-risk group (score < 206.811) and a high-risk group (score ≥ 206.811), with an AUC of 0.788, sensitivity of 70.3%, specificity of 75.7%, and a maximum Youden index of 0.491 (**Figure 4**). Further analysis using the ROC curve was performed to determine the optimal cutoff value for safe margin distance in both risk groups. The findings indicated that in the proximal-margin-positive low-risk group, a margin distance of 2.75cm yielded an AUC of 0.824, with a sensitivity of 54.5%, specificity of 97.9%, and a maximum Youden’s index of 0.524. Conversely, in the proximal-margin-positive high-risk group, a margin distance of 3.85cm resulted in an AUC of 0.813, sensitivity of 73.1%, specificity of 80.0%, and a maximum Youden’s index of 0.531 (**Figure 5**).

## Discussion

The incidence of positive margins in adenocarcinoma of the esophagogastric junction (AEG) has been reported in previous studies to range from 2% to 20%. In our study, we found a positive proximal margin rate of 13.03%, consistent with existing literature (20). The status of surgical margins is a critical prognostic factor for patients with AEG. Research indicates that the five-year survival rate for patients undergoing R0 resection, defined as the complete removal of the tumor with no microscopic residual disease, ranges from 53% to 60%. In contrast, patients who undergo R1 resection, where microscopic residual disease is present, experience a significantly lower five-year survival rate of only 13% to 26% (21–24). The subgroup analysis revealed that while the incidence of positive margins is lower in early-stage AEG patients, these margins retain considerable prognostic significance. Conversely, in late-stage patients, the prognostic impact of positive margins appears diminished, likely due to the increased likelihood of distant metastases that obscure the local effects associated with positive margins (25). Given these findings, clinicians need to strive for the avoidance of positive margins during surgical procedures. Several risk factors have been identified that contribute to the likelihood of proximal margin involvement in patients with AEG. Tumor differentiation plays a critical role, as poorly differentiated adenocarcinomas, specifically undifferentiated and mucinous types, show an increased risk of positive margin status. Additionally, the clinical stage of the tumor is a critical determinant, with higher stages associated with an increased likelihood of margin involvement. The anatomical location of the tumor within the esophagogastric junction (EGJ) is also significant, especially for AEG, which exhibits an increased susceptibility to proximal margin involvement (26). Neoadjuvant therapies, such as chemotherapy and radiotherapy, can induce changes that affect the clarity of surgical margins, complicating surgical outcomes. The technique used during surgery, including the completeness of resection and the skill level of the surgeon, also significantly influences margin status. Moreover, biological characteristics of the tumor, such as vascular and nerve invasion, are linked to an increased risk of positive margin involvement (27, 28). In our study, Borrmann staging, Lauren classification, cT staging, tumor differentiation, and Siewert classification were all significant predictors. The cT staging system, which reflects the depth of tumor infiltration, suggests that deeper tumor invasion correlates with an increased risk of positive margins. Borrmann typing, Lauren typing, and tumor differentiation elucidate the growth patterns and biological characteristics of the tumor, all of which are closely linked to the risk of positive margins. Moreover, Siewert typing, which denotes the anatomical location of the tumor, indicates that patients classified as Siewert type II are at a greater risk for positive proximal margins. The predictive model developed in this study demonstrated improved accuracy when constructed using the SMOTE (Synthetic Minority Over-sampling Technique) dataset, enhancing the area under the curve (AUC) from 0.780 to 0.814. This finding underscores the efficacy of the SMOTE method in addressing data imbalances and suggests that our constructed model exhibits strong predictive capabilities within the test set.

The distance of the proximal margin is closely linked to margin status in patients with AEG; however, a universally accepted standard for margin length has yet to be established. Surgical practices often vary widely, reflecting institutional traditions or individual surgeon preferences rather than evidence-based guidelines (29). Previous investigations indicate that a proximal margin distance of ≤2cm is linked to an elevated risk of margin involvement, particularly in advanced-stage patients (30). Other studies suggest that a gross proximal margin distance of 5 to 12 cm is generally required to minimize the risk of tumor residue and enhance survival rates (31, 32). Specifically, a minimum *in situ* margin length of 5cm is recommended for patients undergoing surgery alone, whereas a proximal margin of 3.8cm was previously considered acceptable (33). The study by Knipper et al. (34) focused on patients who received neoadjuvant therapy and underwent Ivor-Lewis esophagectomy. Their analysis of 660 patients demonstrated that a longer oral resection margin (>33 mm) was associated with significantly improved survival. Specifically, patients with margins exceeding 33 mm had a median survival of 45.0 months (*P*=0.005), compared to those with shorter margins, with lower survival rates. While there is no universal consensus on the exact margin length, several national and international guidelines provide specific recommendations based on tumor depth, histology, and growth pattern. According to the Japanese guidelines, for T2 or deeper tumors, a resection margin of 3 cm is recommended for expansive growth patterns and 5 cm for infiltrative growth patterns (35). Similarly, the ESMO-ESSO-ESTRO guidelines advocate for a 5cm margin for most gastric cancers, increasing to 8 cm for diffuse-type tumors (36). NCCN suggests a minimum margin of 4 cm for T3 and T4 tumors, while AUGIS emphasizes a proximal margin of at least 3.8 cm of normal esophagus for tumors involving the gastroesophageal junction (37, 38). German guidelines recommend proximal margins of 6 cm for the intestinal type and 5–8 cm for the diffuse type, depending on the Lauren classification (39). The use of intraoperative frozen section analysis is commonly recommended when the margin is close or the tumor invades the esophagus, to ensure an R0 resection. These variations highlight the importance of considering tumor biology and the need for individualized surgical planning while emphasizing the role of adequate margin resection to reduce the risk of local recurrence and improve long-term survival. In our analysis, patients were stratified into low-risk and high-risk groups based on proximal margin positivity, with safe margin distances calculated for each group: 2.75cm for low-risk (score < 206.811) and 3.85cm for high-risk (score ≥ 206.811) groups. Notably, Youden’s index was maximized for both cohorts, with AUC values of 0.824 and 0.813, respectively, indicating robust discriminatory ability for proximal margin status. However, this study has several limitations. First, while it focuses on the risk of positive margins in the transabdominal approach, the findings may not be directly applicable to other surgical techniques like ILE or McKeown, which involve different anatomical challenges. Further research is needed to evaluate the risk of positive margins across these approaches. The study also lacks long-term follow-up data, which are essential for understanding the impact of positive margins on survival and recurrence. Moreover, factors like neoadjuvant therapy and surgeon experience were not adequately addressed, though they can influence margin risk. Lastly, as a retrospective study, there is potential for selection bias, limiting generalizability. Prospective studies with standardized protocols are needed to validate these findings.

In conclusion, the nomogram may offer a valuable preoperative tool for assessing the risk of positive proximal margins in AEG patients. While it holds the potential to inform surgical decision-making and help determine appropriate margin distances, further validation in larger and more diverse cohorts is needed to confirm its clinical utility.

## Data Availability

The raw data supporting the conclusions of this article will be made available by the authors, without undue reservation.
